# Mesenchymal stem cells reversibly de-differentiate myofibroblasts to fibroblast-like cells by inhibiting the TGF-β-SMAD2/3 pathway

**DOI:** 10.1186/s10020-023-00630-9

**Published:** 2023-04-25

**Authors:** Ruohao Xu, Miao Wu, Yawen Wang, Chao Li, Lingji Zeng, Yulian Wang, Maozhi Xiao, Xiaomei Chen, Suxia Geng, Peilong Lai, Xin Du, Jianyu Weng

**Affiliations:** Department of Hematology, Guangdong Provincial People’s Hospital (Guangdong Academy of Medical Sciences), Southern Medical University, Guangzhou, Guangdong 510080 People’s Republic of China

**Keywords:** Mesenchymal stem cell, Fibroblast, Myofibroblast, TGF-β1, De-differentiation

## Abstract

**Background:**

Myofibroblasts (MFB), one of the major effectors of pathologic fibrosis, mainly derived from the activation of fibroblast to myofibroblast transition (FMT). Although MFBs were historically considered terminally differentiated cells, their potential for de-differentiation was recently recognized and implied with therapeutic value in treating fibrotic diseases, for instance, idiopathic pulmonary fibrosis (IPF) and post allogeneic hematopoietic stem cell transplantation bronchiolitis obliterans (BO). During the past decade, several methods were reported to block or reverse MFB differentiation, among which mesenchymal stem cells (MSC) have demonstrated potential but undetermined therapeutic values. However, the MSC-mediated regulation of FMT and underlying mechanisms remained largely undefined.

**Method:**

By identifying TGF-β1 hypertension as the pivotal landmark during the pro-fibrotic FMT, TGF-β1-induced MFB and MSC co-culture models were established and utilized to investigate regulations by MSC on FMT in vitro. Methods including RNA sequencing (RNA-seq), Western blot, qPCR and flow cytometry were used.

**Result:**

Our data revealed that TGF-β1 readily induced invasive signatures identified in fibrotic tissues and initiated MFB differentiation in normal FB. MSC reversibly de-differentiated MFB into a group of FB-like cells by selectively inhibiting the TGF-β-SMAD2/3 signaling. Importantly, these proliferation-boosted FB-like cells remained sensitive to TGF-β1 and could be re-induced into MFB.

**Conclusion:**

Our findings highlighted the reversibility of MSC-mediated de-differentiation of MFB through TGF-β-SMAD2/3 signaling, which may explain MSC's inconsistent clinical efficacies in treating BO and other fibrotic diseases. These de-differentiated FB-like cells are still sensitive to TGF-β1 and may further deteriorate MFB phenotypes unless the pro-fibrotic microenvironment is corrected.

**Supplementary Information:**

The online version contains supplementary material available at 10.1186/s10020-023-00630-9.

## Introduction

Pulmonary fibrosis is the primary pathological feature of a series of fatal lung diseases such as idiopathic pulmonary fibrosis (IPF) and post allogeneic hematopoietic stem cell transplantation bronchiolitis obliterans (post-HSCT BO) (Verleden et al. 2016; Wang and Yang 2022). Current paradigms assume these diseases initiate from a pathological activation of MFB differentiation and subsequent deposition of extracellular matrix (ECM), which lead to eventual loss of pulmonary function and death. New drugs for pulmonary fibrosis, such as pirfenidone and nintedanib, were reported to slow the progression of lung fibrosis (Wang and Yang 2022; Costabel et al. 2017; Richeldi et al. 2018). However, these drugs have been proven only to delay the progression of the disease but cannot reverse the natural course of eventual respiratory failure. One of the potential explanations for these dismal results may be that existing therapies fail to reverse established fibrosis or eliminate the pro-fibrotic microenvironment in fibrotic tissue (Bargagli et al. 2019). Effective treatment targeting the established fibrosis thereby avoiding lung transplantation is urgently needed.

Myofibroblasts (MFB) are the core effector and the production workshop of extracellular matrix (ECM) in pathological fibrosis (Kuhn and McDonald 1991). Local FMT-derived MFB cells are the main pathological effector cells of BO (Costabel et al. 2017; Bargagli et al. 2019), with a phenotypic change of the FB to express both fibroblastic and smooth muscle lineages markers such as vimentin and a-smooth muscle actin (a-SMA). These cells appear to have contractile properties as well as enhanced capabilities for matrix production over undifferentiated fibroblasts, resulting in extracellular matrix (ECM) deposition and remodeling of the airway, further leading to tissue scar formation, distortion of the bronchiolar structure, and irreversible loss of function (Verleden et al. 2016). Dysregulation of MFB differentiation/transformation, uncontrolled proliferation and escape of apoptosis are important mechanisms of fibrosis (Bargagli et al. 2019; Kuhn and McDonald 1991; Fortier et al. 2021) involved in the occurrence and development of pulmonary fibrosis. Therefore, MFB-targeted therapies may represent a promising strategy for BO (Fortier et al. 2021).

Mesenchymal stem cells (MSCs) display potent immunomodulatory, anti-inflammatory, anti-fibrosis properties and have been widely used in treating fibrotic disease. Zheng et al. revealed that human adipose-derived MSC intravenous administration alleviates obliterative bronchiolitis (Zheng et al. 2017). Several clinical studies have revealed that MSC infusion represents an effective and safe treatment option for post-HSCT BO (Zhou et al. 2010; Chen et al. 2019). Despite the promising results of MSC in treating BO, the duration of MSC-associated efficacies are unsatisfactory. Detailed interactions between infused MSC with residual MFB cells remained largely uninvestigated. According to our previous report, the MSC infusion demonstrated certain efficacies in alleviating skin, mouth, eye and gastrointestinal tract syndromes in patients with refractory chronic graft-versus-host disease. However, no treatment response was seen concerning BO syndromes after the MSC infusion (Weng et al. 2010). Elucidating the MSC-mediated regulatory effects on the FMT process may help elucidate heterogeneous clinical outcomes, thus bringing new strategies in treating BO.

This present study included three major parts of work to address these issues. RNA-seq datasets on fibrotic lung FB samples were extracted to identify central events during the FMT process. MFB induction and umbilical cord-derived MSC (uMSC) co-culture models were then established and utilized to investigate regulations by uMSC on FMT in vitro. At last, RNA-seq, qPCR, Western blot, and flow cytometry were used to investigate intercellular regulations of FMT. Our findings provide cognitive schemas for the uMSC-mediated regulations on FMT, which may help facilitate the utilization of uMSC in treating BO and other lung fibrotic diseases.

## Methods

### Online high-throughput RNA sequencing datasets and data collection

After literature screening, three high-throughput RNA-seq datasets of FB samples from patients with lung fibrosis (IPF) and healthy donors (HD) were downloaded from the National Center for Biotechnology Information (NCBI) GEO database (https://www.ncbi.nlm.nih.gov/geo/) and analyzed. The GSE185492 dataset included 24 lung FB samples from either IPF patients (n = 12) or HD (n = 12). Lung FB samples were further classified as the apex group (n = 12) and base group (n = 12) according to the site where biopsies were performed. Another dataset GSE180415 included 9 primary FB samples from the upper lobe lung from IPF patients (n = 5) and HD (n = 4). To further identify invasive signatures in IPF-FB samples, which were reported to be associated with fibrosis progression (Geng et al. 2019), transcriptomes of both invasive IPF-FB (n = 9) and non-invasive IPF-FB (n = 9) (GSE118933) samples were profiled and analyzed. Details of each online dataset and corresponding sequence platforms were listed (Additional file 1: Table S1).

### Cell culture

MRC-5 normal human lung FB cells were purchased from American Type Culture Collection. FB cells were cultured using DMEM supplemented with 10% fetal bovine serum (FBS) containing 100 units/mL penicillin and 100 ug/mL streptomycin (both from Gibco). For MFB differentiation, FB was serum-starved in FBS-free DMEM overnight and stimulated with 10 ng/mL TGF-β1 (PeroTech, USA) for 48 h to induce MFB differentiation. For transwell co-culture of MFB with uMSC, the adherent TGF-β1-induced MFBs were added with an additional uMSC feeder layer and co-cultured indirectly for 24 h before subsequent RNA-seq and functional assays. According to previous reports, the MFB differentiation is induced time-dependent and remains stable for up to 2–4 days after a single dose of TGF-β1 treatment (Hecker et al. 2011; Vaughan et al. 2000; Thannickal et al. 2003). To simulate the microenvironment where TGF-β1 is persistently elevated in IPF/BO, a TGF-β1 concentration of 10 ng/mL was also maintained in our non-contact transwell co-culture system.

### Cell preparation and RNA-seq

Total RNA of FB, MFB, and FB-like cells were extracted using RNEasy Kit (Qiagen, Germantown, MD). RNA integrity was assessed using the RNA Nano 6000 Assay Kit of the Bioanalyzer 2100 system (Agilent Technologies, CA, USA). mRNA was purified from total RNA and fragmented, and cDNA was synthesized. cDNA fragments in 370 ~ 420 bp were extracted, and library fragments were purified with the AMPure XP system (Beckman Coulter, Beverly, USA). After the quality control process, the library preparations were sequenced on an Illumina Novaseq platform, and 150 bp paired-end reads were generated. All experiments were performed in triplication.

### Data analysis

Gene expression matrices of GSE185492 and GSE180415 were obtained from the NCBI GEO database. For GSE118933, fastq files sequencing raw data were downloaded and aligned to the human genome (GRCh38) by HISAT2 (version 2.1.0), and raw counts were calculated (Kim et al. 2015). Differentially expressed genes were calculated by DESeq2 (Love et al. 2014) (R package version: 1.18.1). Genes were determined to be differentially expressed based on an adjusted P-value < 0.05. Unsupervised clustering analysis was performed using facotextra (R package version: 1.05). Heatmaps were plotted using gplots (R packages, version 3.01). Gene set enrichment analysis was performed using eVITTA as described (Cheng et al. 2021). Enriched gene ontology analysis (G.O.) was performed by ClusterProfiler (Yu et al. 2012) (R package version 3.6.0).

### RT-qPCR and Western Blot

Total RNA was extracted using Trizol (Gibco, USA), and cDNA was synthesized using Evo M-MLV RT Kit (Accurate Biotechnology, China) following manufacturers' instructions. Relative gene expression was measured relative to the endogenous gene GAPDH using the delta CT method. The ID and primer sequence of detected genes were listed (Additional file 1: Table S2).

Cell samples were lysed with RIPA buffer (Gibco, USA), and proteins were resolved by SDS-PAGE using gradient gels (4%-20%) and electroblotted onto PVDF membranes es (Millipore, German). Then membranes were probed with rabbit anti-GAPDH (1:1000; Abcam, USA), rabbit anti-α-SMA (1:1000; Abcam, USA), rabbit anti-COL1A1 (1:1000; Abcam, USA), rabbit anti-COL3A1 (1:1000; Abcam, USA) and rabbit anti-fibronectin (1:1000; Abcam, USA) at 4 °C overnight, followed by three rinses with Tris-buffered saline/Tween (TBST) and incubated at a 1:5000 dilution of goat anti-rabbit IgG secondary antibodies (CST, China). GAPDH was used as the loading control.

### Cell viability/proliferation and apoptosis detection

Cell Counting Kit-8 (CCK-8, Beyotime, China) was used to estimate cell viability/proliferation. Resuspended cells were adjusted to 5 × 10^3^/well and laid in 96-well cell culture plates with a 100 μL medium. Then 10 μL of CCK-8 to each well was added and incubated for 2 h. The absorbance of each well was then measured at 450 nm using a microplate reader (Bio-Rad, CA, USA). An Annexin V/PI apoptosis detection kit (Sigma, USA) was used for apoptosis detection. A flow cytometry (FACS Canto II; B.D. Biosciences, San Jose, CA, USA) was used to determine the population and apoptosis rate. Cells were resuspended in Hank's balanced saline solution supplemented with 2% FBS and 0.1 mM EDTA. According to the manufacturer's protocol, the apoptosis rates were quantified by staining cells with FITC-Annexin V and PI in the dark at room temperature.

### Statistics

Data are displayed as mean ± SEM. All experiments were performed at least three or more times. Student's t-test (2-sided) was used to compare the two groups' differences. One-way or 2-way ANOVA analysis followed by the Turkey-Kramer test was utilized for multiple comparisons. Statistically, significance was set at P < 0.05. GraphPad Prism 7.0 software and R were used for statistical analysis.

## Results

### Identification of transcriptomic signatures in pro-fibrotic FB from patients with lung fibrosis

**Fig. 1:**
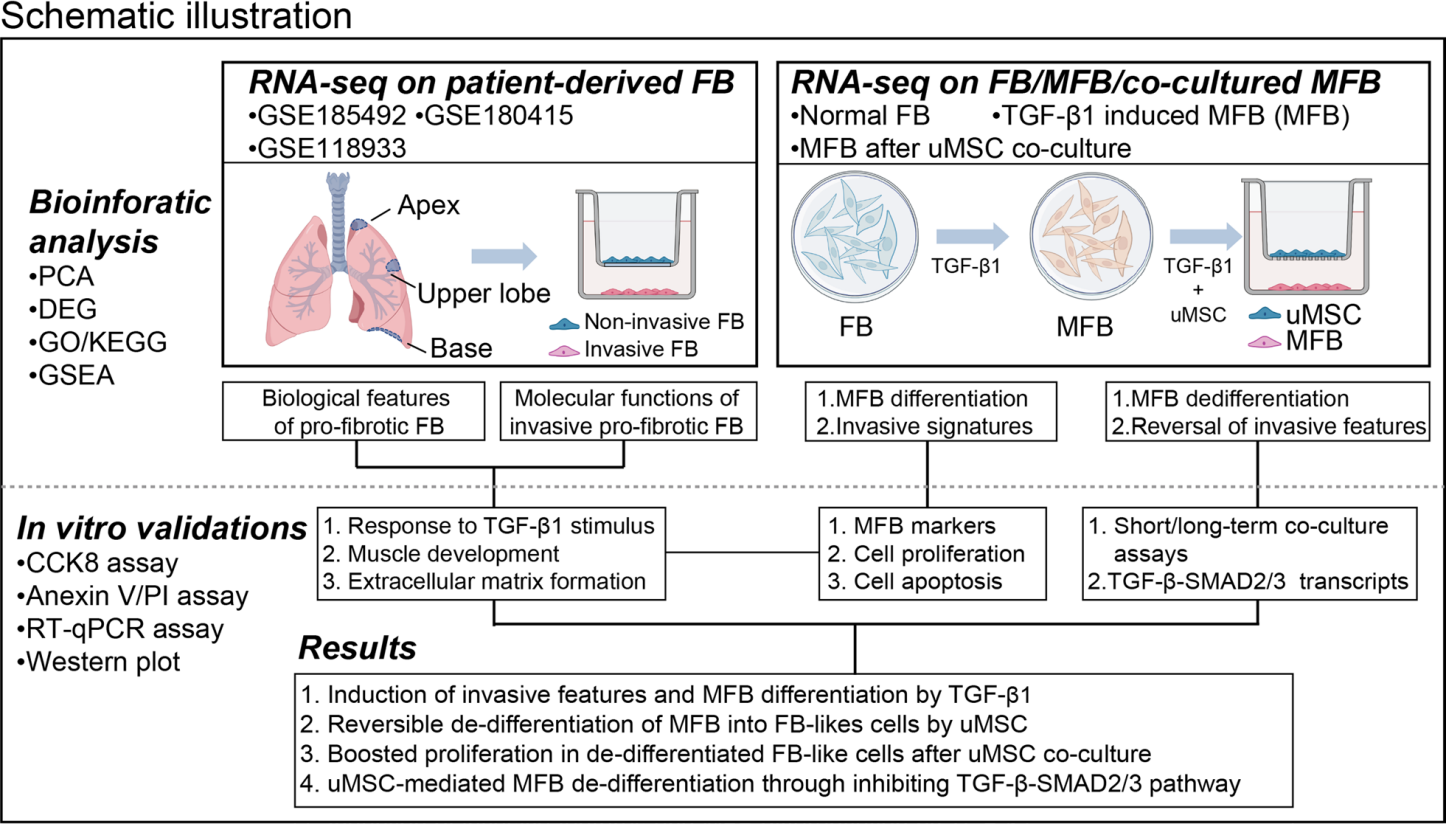
Schematic illustration of the present study. *PCA* principal component analysis, *DEG* differential expressed gene, *GO* gene ontology analysis, *KEGG* Kyoto Encyclopedia of Genes and Genomes Atlas, *GSEA* gene set enrichment analysis, *RNA-seq* RNA sequencing, *uMSC* Umbilical cord-derived mesenchymal stem cells, *TGF-β1* transforming growth factor-β1, *FB* fibroblast, *MFB* myofibroblast

**Fig. 2:**
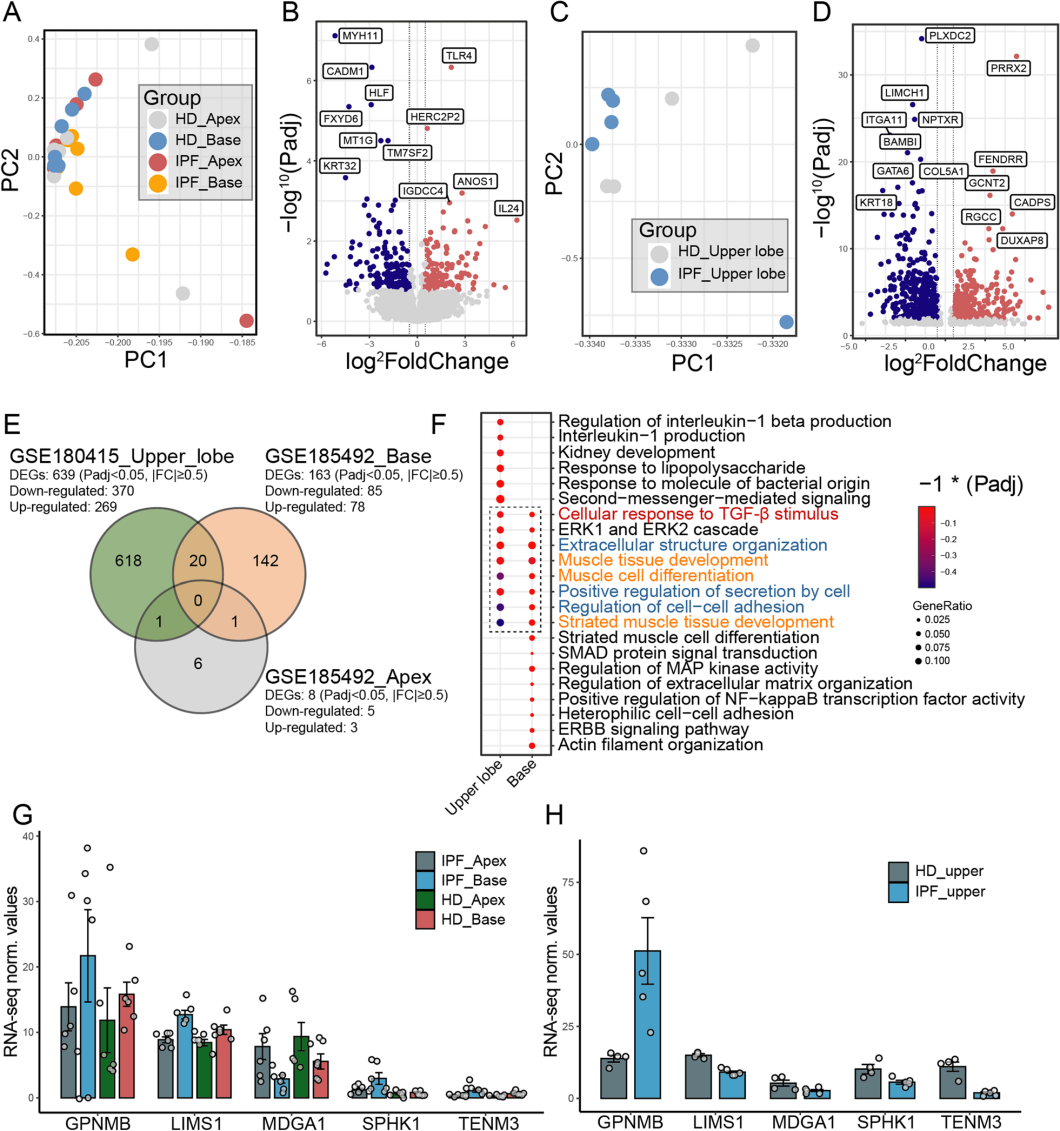
Identifications of transcriptomic profiles of lung FB samples from patients with IPF and healthy donors (HD). **A** Principal component analysis on IPF/HD-derived FB samples from the apex (n = 12) and basal lung sites (n = 12, GSE185492). **B** Volcano plot displaying identified DEGs in IPF-FB compared with HD-FB from the basal lung site (GSE185492) by log2 foldchange (x-axis) and adjust P-value (y-axis). Blue dots represent DEGs significantly down-regulated; red dots represent DEGs significantly upregulated; grey dots represent genes not significantly regulated. **C** Principal component analysis on IPF/HD-derived FB samples from the upper lobe lung site (n = 9, GSE180415). **D** Volcano plot displaying identified DEGs in IPF-FB compared with HD-FB from the upper lobe lung site (GSE180415) by log2 foldchange (x-axis) and adjust P-value (y-axis). Blue dots represent DEGs significantly down-regulated; red dots represent DEGs significantly upregulated; grey dots represent genes not significantly regulated. **E** Venn diagrams depicting the number of DEGs specifically or mutually regulated in IPF-FB samples from different lung anatomical sites. **F** Enriched analysis by EGO using DEGs identified in IPF-FB samples from upper lobe lung and basal lung sites. **G**, **H** RNA-seq expression normalized value (fragments per kilobase of exon model per million mapped fragments, FPKM) for transcripts encode for genes related to response to TGF-β1 stimulus and muscle tissue development in each RNA-seq dataset

MFB originates predominantly from FB through the activation of fibroblast-myofibroblast transition (FMT) (Saito et al. 2018). However, molecular and functional regulations in FMT remained poorly investigated. By analyzing RNA-seq datasets of patient-derived FB samples from patients with lung fibrosis (IPF) and healthy donors (HD), we first identified the pathogenic signatures of FB under a pro-fibrotic transformation (Fig. 1, Additional file 1: Table S1). Unsupervised principal component analysis (PCA) revealed a major transcriptomic inconsistency among pro-fibrotic FB samples and normal samples (GSE185492) (Fig. 2A). Similar transcriptomic inconsistency was also captured in pro-fibrotic FB samples from another anatomical lung site (GSE180415) (Fig. 2C). By performing gene expressing analysis using a cutoff threshold of Padj value < 0.05 and log^2^foldchange > 0.5, a total of 163 differentially expressed genes (DEGs) were identified in IPF-FB from the basal lung site (78 up-regulated and 85 down-regulated) (Fig. 2B). Meanwhile, 639 DEGs (269 up-regulated and 370 down-regulated) were identified in IPF-FB from the upper lobe lung site (Fig. 2D). In accordance with the HRCT (high-resolution CT) based categories, which indicate limited involvement in the apex IPF lung site (Raghu et al. 2022), little difference was found between IPF-FB and HD-FB in the apex lung site, with only 8 DEGs identified (data not shown).

To ensure the accuracy and sensitivity of the analysis, subsequent bioinformatics analysis was performed exclusively using FB samples from the basal and upper lobe lung sites. Among identified DEGs in IPF-FB from each dataset, only 20 DEGs were shared by IPF-FB from the basal lung (3.1%) and upper lobe lung (12.3%), and none of the DEGs were mutually identified in all three lung sites (Fig. 2E). Those shared DEGs identified in IPF-FB samples mainly indicated invasive signatures in fibrotic diseases (GPNMB, TENM3) (Palisoc et al. 2022; Athwal et al. 2018; Murakami et al. 2010), cell focal adhesion (LIMS1) (Sandfort et al. 2010; Stanchi et al. 2009), cell cycle (MDGA1) (Lu et al. 2008), and angiogenesis (SPHK1) (Wang et al. 2021) (Fig. 2G, H). Based on the DEGs sets, 49 and 21 EGO (enriched gene ontology) terms were enriched in IPF-FB from the upper lobe and basal lung sites (Padj < 0.05), while no term was enriched in IPF-FB from the apex lung site (Fig. 2F). These EGO terms mainly reflected the enrichment of cellular functions, including muscle tissue development (Fig. 2F, orange) and ECM remodeling (Fig. 2F, blue). Previous studies emphasize the pivotal role of TGF-β1 in initiating and maintaining MFB and fibrosis (Saito et al. 2018; Aschner and Downey 2016). Similar to these reports, our analysis also revealed a prominent signature reflecting a cellular response to TGF-β1 (Fig. 2F, red), which underlined the potential contributions of TGF-β1 to FMT pathological signatures observed.

### TGF-β1 induced invasive signatures and initiated MFB differentiation in normal FB

**Fig. 3:**
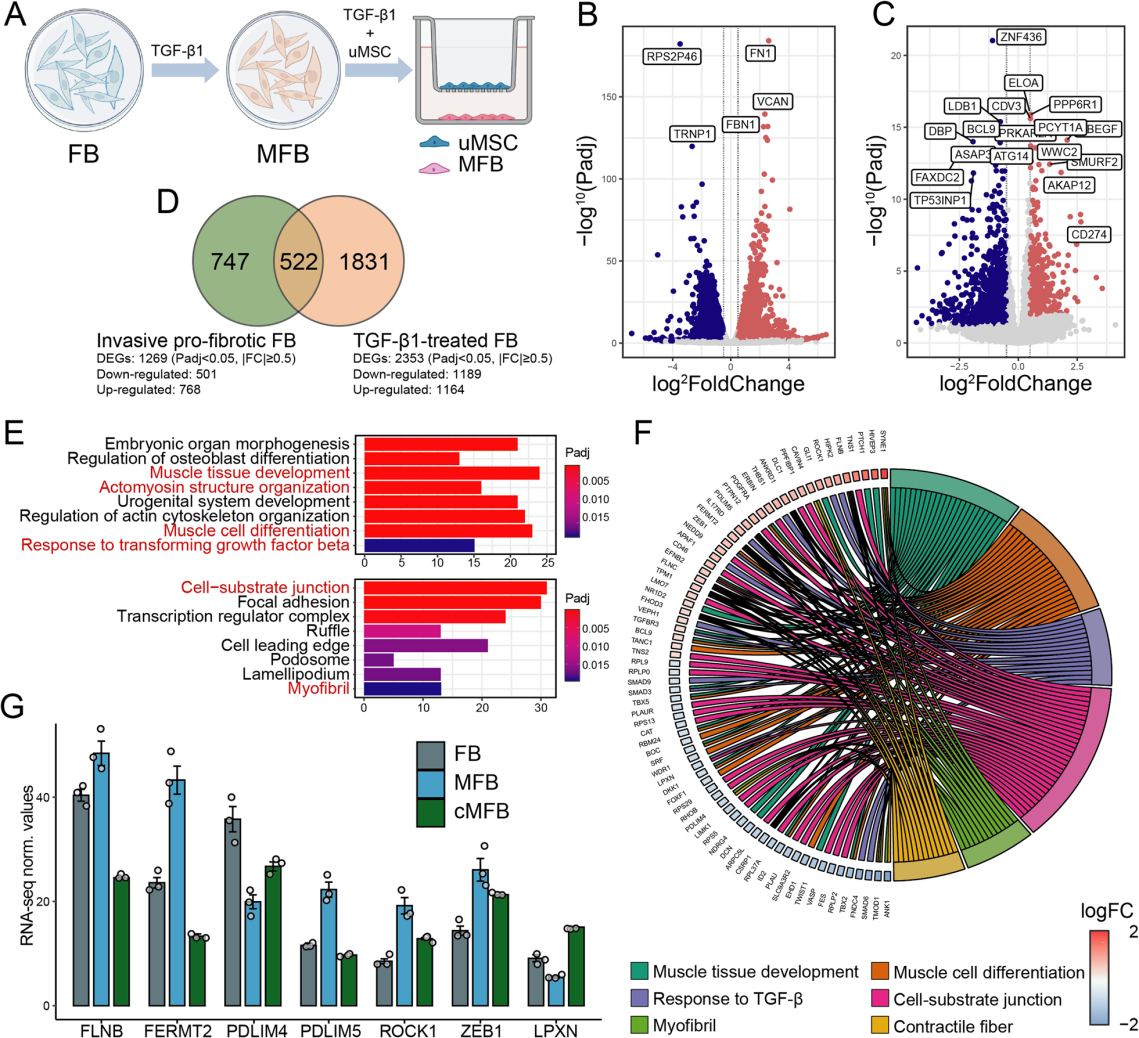
In vitro administration of TGF-β1 induced invasive IPF-FB signatures in normal lung FB samples. **A** Schematic representation of in vitro TGF-β1 induction and subsequent uMSC co-culture models. Normal lung FB was treated with ether 10 ng/mL of TGF-β1 or DMSO before being subjected to subsequent RNA-seq and in vitro assays. **B** Volcano plot displaying identified DEGs in MFB compared with untreated FB by log2 foldchange (x-axis) and adjust P-value (y-axis). **C** Volcano plot displaying identified DEGs in invasive IPF-FB (n = 9) compared with non-invasive IPF-FB (n = 9, GSE118933) by log2 foldchange (x axis) and adjust P value (y axis). **D** Venn diagrams depicting the number of DEGs specifically or mutually regulated in invasive IPF-FB and MFB samples. **E** Enriched gene ontology analysis (EGO) concerning biological process (BP), molecular function (MF), and cellular component (CC) of DEGs identified in MFB (MFB vs. FB). **F** Circos plot displaying the most significantly enriched EGO terms and related DEGs participating in each process. Symbols of DEGs are displayed on the left side of the graph with their fold change value mapped by color (red represents up-regulation and blue represents down-regulation). **G** RNA-seq expression normalized value (FPKM) for transcripts encode for genes related to TGF-β signaling, muscle/fibrin development in FB, iMFB, and co-cultured MFB

To evaluate the impacts of identified pro-fibrotic factor TGF-β1 on the cellular functions and transcriptomes of normal lung FB, MRC-5 human lung FB cells were treated with TGF-β1 or DMSO for 48 h and subjected to RNA-seq (Fig. 3A). The TGF-β1 treatment resulted in 2353 DEGs (1164 up-regulated and 1189 down-regulated) in normal FB (Fig. 3B). Similar to that seen in FB samples derived from patients with lung fibrosis, these TGF-β1-treated FB cells displayed initiation of MFB differentiation and ECM remolding (Fig. 3E, red).

Interestingly, TGF-β1 also readily induced pathological invasive transcriptomes, which could be seen in that patient-derived invasive fibrotic FB samples. By comparing transcriptomes in invasive FB samples from the GSE118933 dataset (Fig. 3C), up to 41.1% (522/1269) of DEGs identified in invasive IPF-FB were simultaneously induced by TGF-β1 in normal FB samples (Fig. 3D). The most significantly enriched EGO terms and foldchanges of these overlapped DEGs were indicated activations in functions including "contractile fiber" (GO:0043292), "myofibril" (GO:0030016), and "cell-substrate junction" (GO:0030055) in TGF-β1-treated FB (Fig. 3F). Following these EGO terms, up-regulated expression of TGF-β1 signaling transcripts (Turner et al. 2019; Baudier et al. 2018; Zheng et al. 2022) (FLNB, 1.51-foldchange; ROCK1, 1.17-foldchange) and muscle/fibrin development (Karlsen et al. 2022; Yan et al. 2021) (FERMT2, 0.87-foldchange; PDLIM5, 0.94-foldchange) were validated by fragments per kilobase of exon model per million mapped fragments (FPKM) (Fig. 3G). Therefore, these results suggested that TGF-β1 may confer an invasive transcriptomic phenotype in normal FB.

**Fig. 4:**
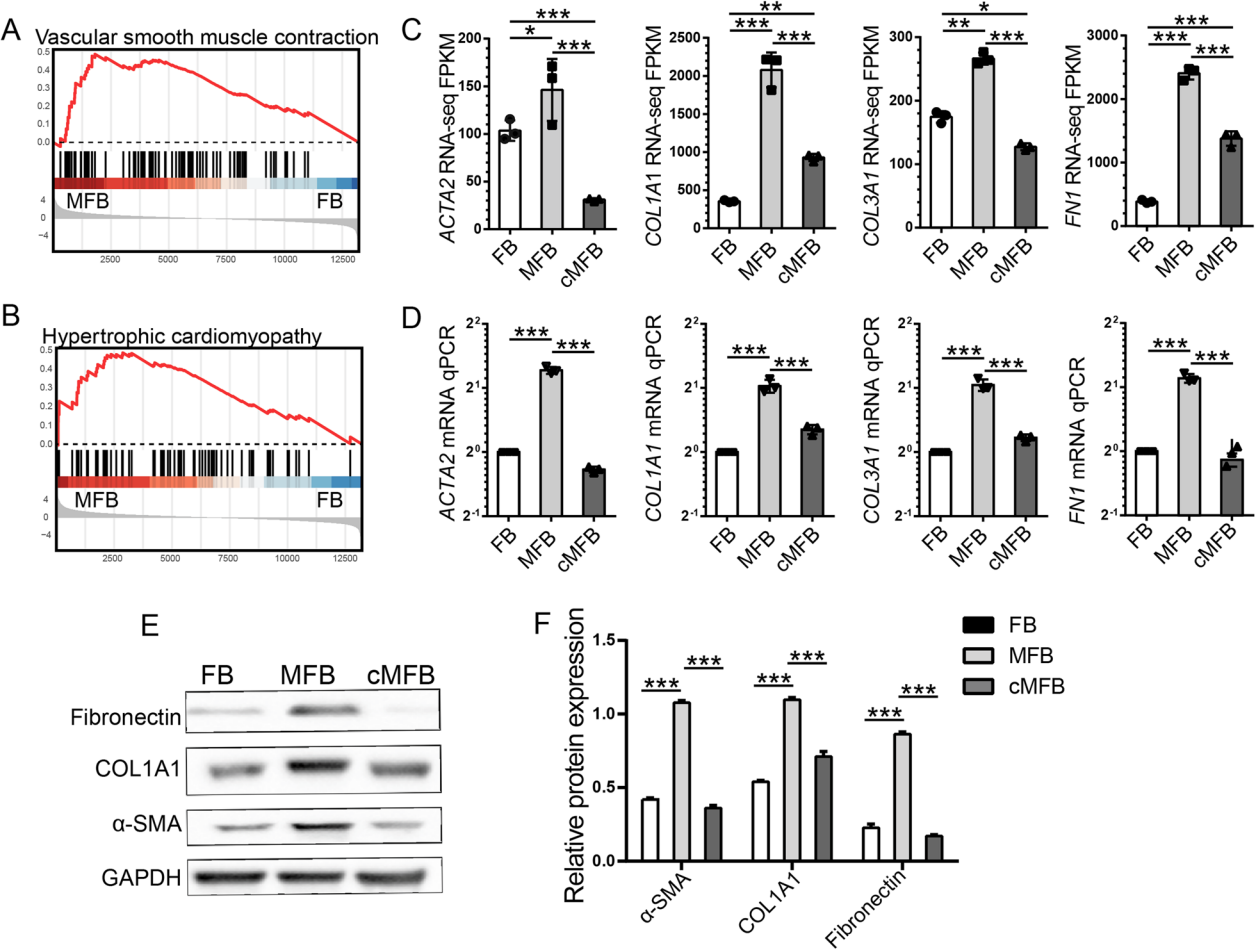
Co-culturing with uMSC inhibited TGF-β-induced expression of MFB-specific markers. **A**, **B** Gene set enrichment analysis (GSEA) indicates activation of vascular smooth muscle contraction and hypertrophy cardiomyopathy functions in TGF-β1-induced MFB. **C**, **D** RNA-seq gene expression (FPKM) and qPCR validations for MFB markers, including *ACTA2* (encoding for a-SMA), *COL1A1*, *COL3A1,* and *FN1* in FB, MFB, and co-cultured MFB samples. **E**, **F** Western blot assays on fibronectin, COL1A1, and a-SMA proteins in FB, MFB, and co-cultured MFB samples. FPKM, fragments per kilobase of exon model per million mapped fragments. * < 0.05; **P < 0.01; ***P < 0.001

To more precisely annotate biological entities induced by TGF-β1, gene set enrichment analysis (GSEA) was performed and revealed identical enrichment of smooth muscle contraction and pathological muscle tissue development (Fig. 4A, B). A significant up-regulation of established MFB markers (Fortier et al. 2021; Saito et al. 2018; Aschner and Downey 2016), including ACTA2 (0.5-foldchange up-regulated), COL1A1 (2.55-foldchange up-regulated), COL3A1 (0.61-foldchange up-regulated) and FN1 (2.64-foldchange up-regulated) was seen in TGF-β1-treated FB and was validated by RT-qPCR and Western blot assays (Fig. 4D–F). These findings provided a rationale for the administration of TGF-β1 to initiate MFB differentiation in normal FB. They could be utilized as a model for further studies on the regulatory effects of uMSC on FMT.

### Short-term co-culturing of MFB with uMSC led to reversible de-differentiation into FB-like cells

To investigate potential uMSC-mediated regulatory effects on the pro-fibrotic FMT, non-contact transwell co-culture assays of MFB with uMSC were performed. The MFB differentiation is induced time-dependent and remains stable for up to 2–4 days after a single dose of TGF-β1 treatment (Hecker et al. 2011; Vaughan et al. 2000; Thannickal et al. 2003). To simulate the microenvironment where TGF-β1 is persistently elevated in IPF/BO, TGF-β1 at the concentration of 10 ng/mL was also maintained in our MFB-MSC non-contact transwell co-culture system (Fig. 3A).

Samples of normal FB (n = 3), MFB (n = 3) and co-cultured MFB (n = 3) were harvested and then subjected to high-throughput RNA sequencing (RNA-seq) and functional assays. We first used flow cytometry to investigate the expression of surface antigens of those untreated FB, MFB and FB-like cells. Results indicated that those FB, MFB and co-cultured MFB samples shared a similar expression of mesenchymal-associated surface antigens at the protein level (Additional file 1: Fig. S1), which collaborates with previous reports (Hajizadeh-Tafti et al. 2022; Lupatov et al. 2015). Since MSC is a group of cellular properties with secretory functions and shares a common mesenchymal origin with FB and MFB, we next sought to investigate the potential assimilation effect on the co-cultured MFB. After directly comparing the transcriptomes of co-cultured MFB with our previously published data of normal MSCs (Xu et al. 2021), our results ruled out the potential assimilation effect by confirming the lack of expression of MSC-specific transcripts in these co-cultured cells (Additional file 1: Fig. S2).

**Fig. 5:**
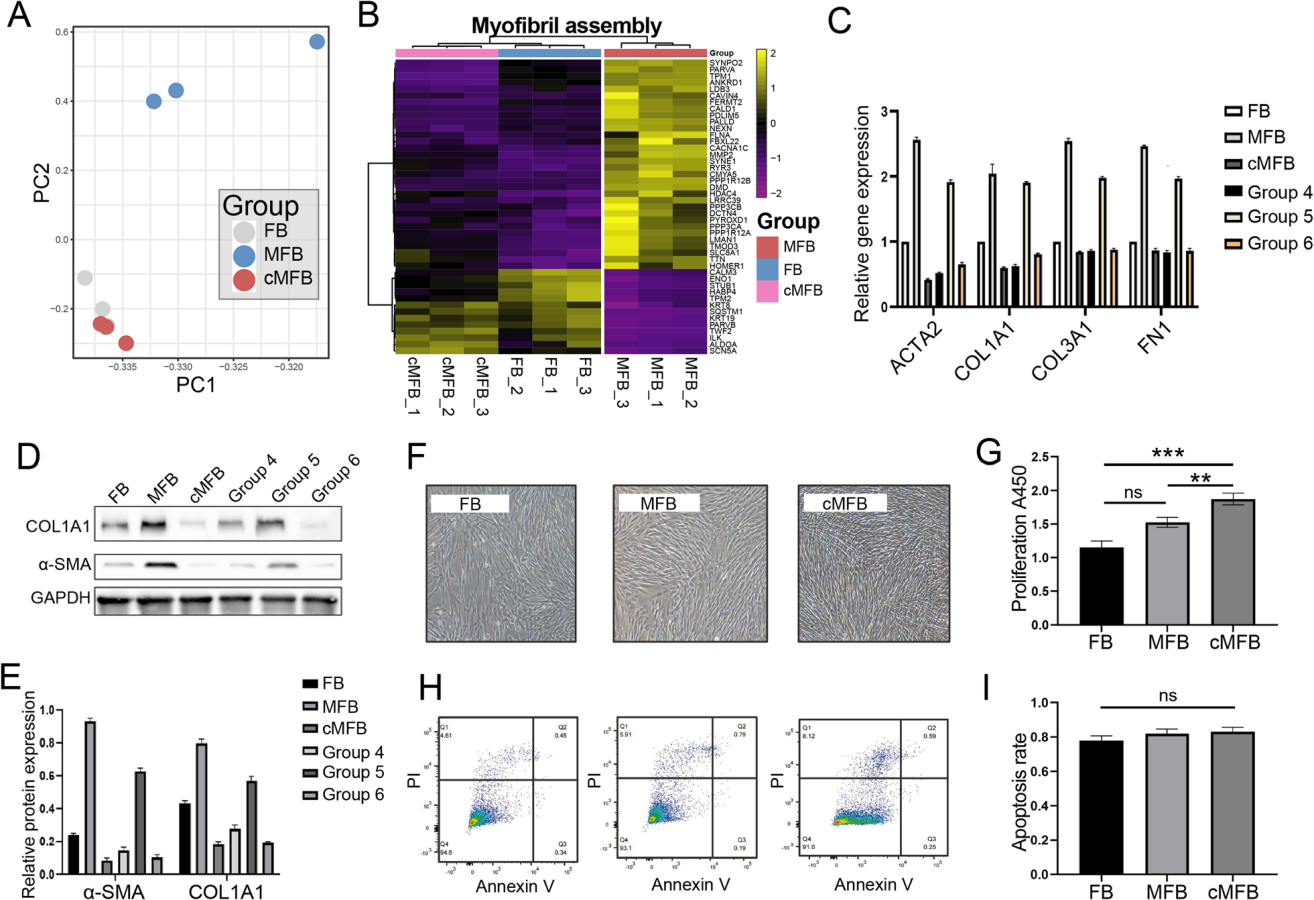
Identification of the cellular properties and reversibility of uMSC-mediated MFB de-differentiation. **A** Principal component analysis (PCA) on transcriptomes of FB, MFB, and co-cultured MFB samples. **B** Unsupervised clustering heatmap of 45 gene-panel involved in myofibril assembly on FB, MFB, and co-cultured MFB samples. **C** RT-qPCR assays on the expression of *ACTA2* (encoding for a-SMA), *COL1A1*, *COL3A1,* and *FN1* in cells from the 6 group settings: (1) Untreated FB; (2) MFB; (3) Short-term co-cultured MFB (FB-like cells); (4) Long-term co-cultured MFB; (5) FB-like cells treated with TGF-β1 without uMSC feeder layer; (6) TGF-β1-treated FB-like cells with additional uMSC feeder layer. **D**, **E** Western blot assays on FN1, COL1A1, and a-SMA proteins in cells from the 6 group settings: (1) Untreated FB; (2) MFB; (3) Short-term co-cultured MFB (FB-like cells); (4) Long-term co-cultured MFB; (5) FB-like cells treated with TGF-β1 without uMSC feeder layer; (6) TGF-β1-treated FB-like cells with additional uMSC feeder layer. **F**, **G** The proliferation of FB, MFB, and co-cultured MFB by light microscopy and CCK-8 assays. **H**, **I** Apoptosis FB, MFB and co-cultured MFB by Annexin V/PI assays. * < 0.05; **P < 0.01; ***P < 0.001

Next, unsupervised principal component analysis (PCA) was performed. As expected, huge transcriptomic heterogeneity was revealed between normal FB and MFB. Interestingly, co-culturing with uMSC significantly diminished this transcriptomic heterogeneity. Altered transcriptomes committed by TGF-β1 treatment in MFB were largely rescued by uMSC, resembling that observed in untreated FB samples (Fig. 5A). Myofibril assembly represents a characteristic function of MFB, while the activity of this function is inhibited in normal FB (Rodriguez et al. 2019). Illustrated by an unsupervised clustering heatmap of 45 gene-panel involved in myofibril assembly, most up-/down-regulated genes observed in MFB samples were significantly rescued after co-culturing with uMSC. Being preferentially clustered with FB samples, these co-cultured MFB cells resembled normal FB (Fig. 5B). However, our further analysis revealed that these FB-like cells still showed certain differences at the transcriptome level compared with untreated FB (Additional file 1: Fig. S3). Therefore, these data revealed that uMSC de-differentiates TGF-β1-induced MFB into a group of FB-like cells.

Next, to investigate whether this uMSC-mediated MFB dedifferentiation is durable or reversible, RT-qPCR and Western blot on MFB markers were performed using cells from the following 6 group settings: (1) Untreated FB; (2) MFB; (3)Short-term co-cultured MFB (FB-like cells); (4)Long-term co-cultured MFB; (5) FB-like cells treated with TGF-β1 without uMSC feeder layer; (6) TGF-β1-treated FB-like cells with additional uMSC feeder layer. As shown by RT-qPCR, the expression of MFB markers (*ACTA2*, *COL1A1*, *COL3A1,* and *FN1*) in those FB-like cells was significantly down-regulated when co-culturing with uMSC in the presence of TGF-β1 (Fig. 5C, group 3). However, after withdrawal from the uMSC feeder layer, these de-differentiated FB-like cells remained sensitive to TGF-β1 and were immediately re-induced into MFB (Fig. 5C, group 5). Western blot assays on a-SMA and COL1A1 protein further validated the dynamics of these MFB markers (Fig. 5D, E). Cellular proliferation and apoptosis of each group of FB-derived progenies were also determined. Revealed by light microscopy and CCK-8 assays, the proliferation of FB-like cells was significantly boosted compared with that in normal FB and MFB group (1.87 ± 0.05 vs1.12 ± 0.04, P < 0.001), while the proliferation was not significantly increased in the MFB group compared with that in the FB group (Fig. 5F, G). The apoptosis rate was not significantly different among the group settings (Fig. 5H, I).

### uMSC de-differentiated TGF-β1-induced MFB by selectively inhibiting the TGF-β-SMAD2/3 signaling pathway

**Fig. 6:**
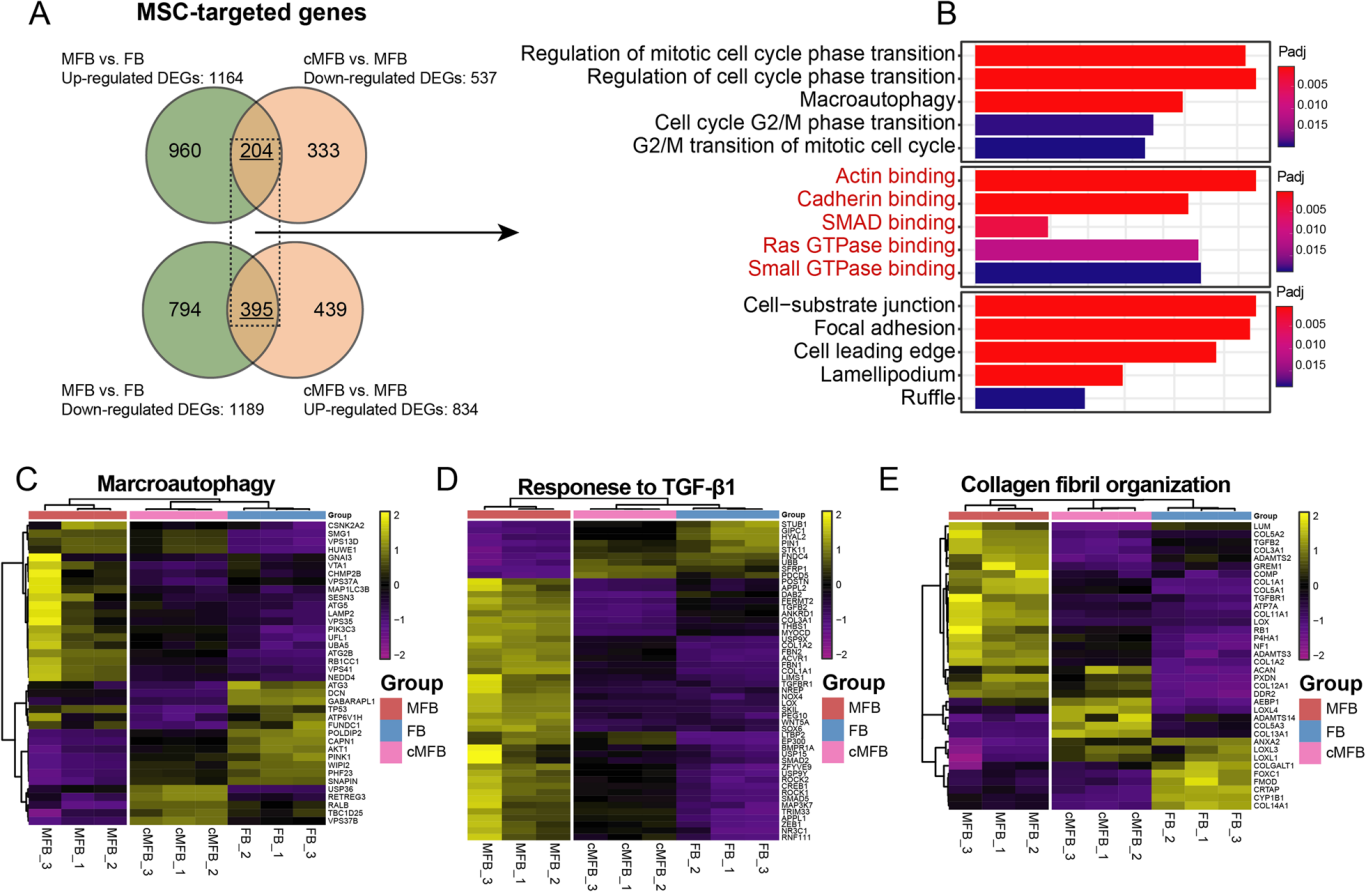
Annotation of the targeted genes during uMSC-mediated MFB de-differentiation. **A** Venn diagrams depicting the number of DEGs regulated in MFB (MFB vs. FB) and then reversely regulated in co-cultured MFB (co-cultured MFB vs. MFB). **B** Enriched gene ontology analysis (EGO) concerning biological process (BP), molecular function (MF), and cellular component (CC) using the targeted DEGs. **C**–**E** Unsupervised clustering heatmap on genes participating in macroautophagy, response to TGF-β1, and collagen fibril organization in FB, MFB, and co-cultured MFB samples

To further elucidate the molecular transductors of MSC-mediated de-differentiation in MFB, those selectively targeted transcripts were identified, defined as DEGs significantly regulated in MFB (MFB vs. FB) and then reversely regulated after co-culturing with uMSC (FB-like cells vs. MFB). Generally, among 2353 DEGs identified in MFB (MFB vs. FB), a total of 599 DEGs were reversely regulated after co-culturing with uMSC (FB-like cells vs. MFB), thus were considered as transcriptomic targets in uMSC-mediated dedifferentiation (Fig. 6A). Annotation on these genes revealed significant enrichment for a range of molecular functions (MF), including actin-binding (GO:0003779), cadherin binding (GO:0045296), and SMAD binding (GO:0046332) (Fig. 6B, red). For functional features, the expression pattern of macroautophagy (Fig. 6C), response to TGF-β1 (Fig. 6D), and collagen fibril organization (Fig. 6E) were also significantly regulated in FB-like cells.

**Fig. 7:**
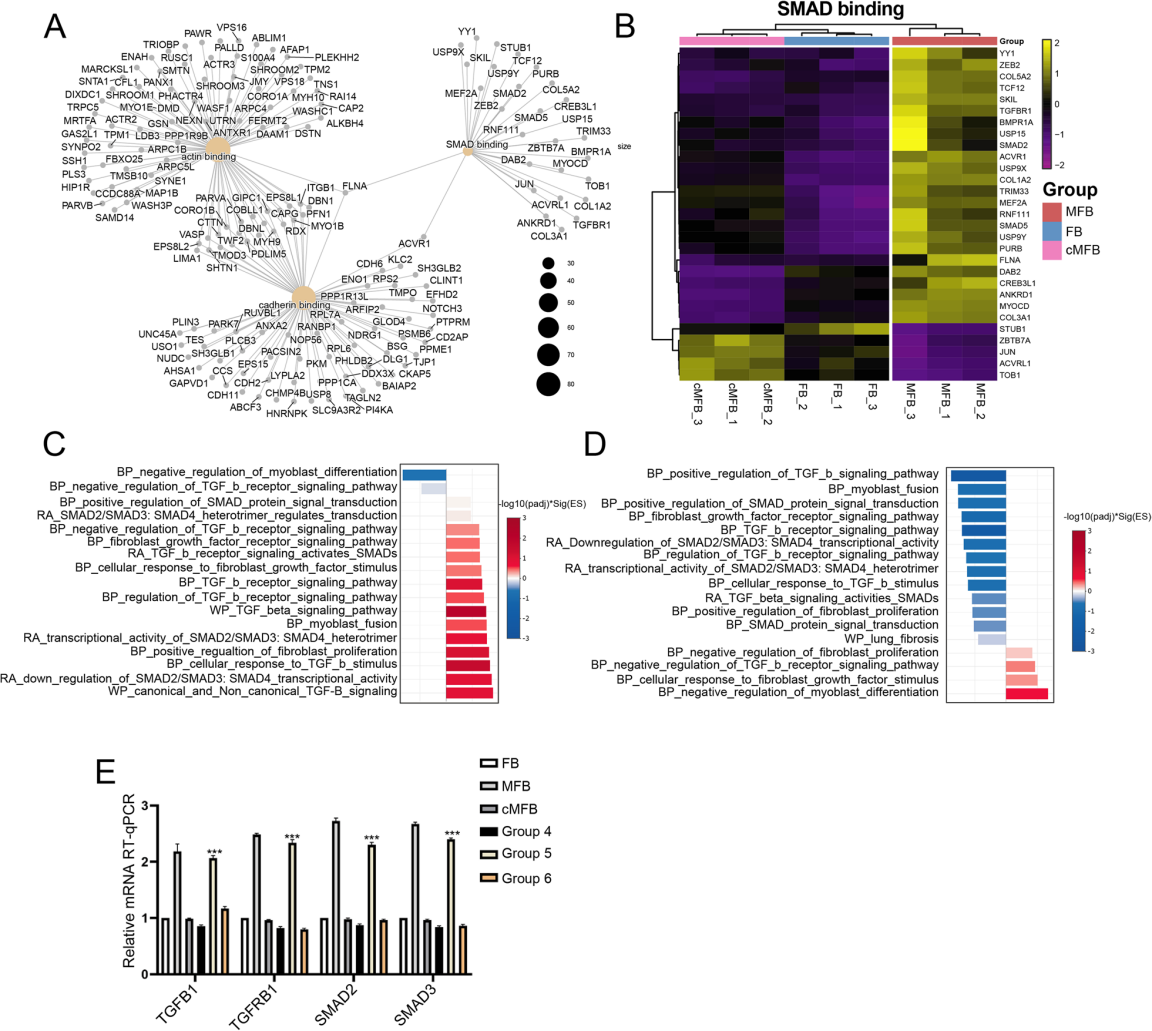
Reversible modulations on the TGF-β-SMAD2/3 signaling pathway in the uMSC-mediated MFB de-differentiation. **A** Network illustration of actin-binding, cadherin binding, SMAD binding, and associated DEGs. **B** Unsupervised clustering heatmap on genes participating in SMAD binding in FB, MFB, and co-cultured MFB samples. **C** GSEA analysis on regulated genes identified in MFB (MFB vs. FB). **D** GSEA analysis on regulated genes identified in co-cultured MFB (co-cultured MFB vs. MFB). **E** RT-qPCR assays on the expression of *TGFB1*, *TGFBR1*, *SMAD2* and *SMAD3* in cells from the 6 group settings: (1) Untreated FB; (2) MFB; (3) Short-term co-cultured MFB (FB-like cells); (4) Long-term co-cultured MFB; (5) FB-like cells treated with TGF-β1 without uMSC feeder layer; (6) TGF-β1-treated FB-like cells with additional uMSC feeder layer

TGF-β1 directly activates Smad signaling, for instance, the Smad2/3 signaling, whose pathological activation was correlated with fibrosis development (Hu et al. 2018). Network analysis of the top 3 enriched MF terms confirmed a significant enrichment of SMAD binding function and indicated a range of associated DEGs (Fig. 7A). Most of these SMAD-involving DEGs were then confirmed to be significantly up-regulated in MFB and then rescued after co-culturing with uMSC (Fig. 7B). By performing a more integrated analysis that incorporates both terminology and foldchange of each gene, GSEA analysis confirmed that TGF-β1 treatment activated a wide range of SMAD signaling together with MFB-associated functions (Fig. 7C). Following the results from the functional annotation of genes targeted by uMSC, GSEA also revealed that uMSC rescued the majority of SMAD signaling functions, among which the SMAD2/3 signaling is predominantly down-regulated (Fig. 7D). These data prove that the uMSC-mediated MFB de-differentiation is conducted through selective regulation of the TGF-β-SMAD2/3 signaling within the MFB during the de-differentiation process.

**Fig. 8:**
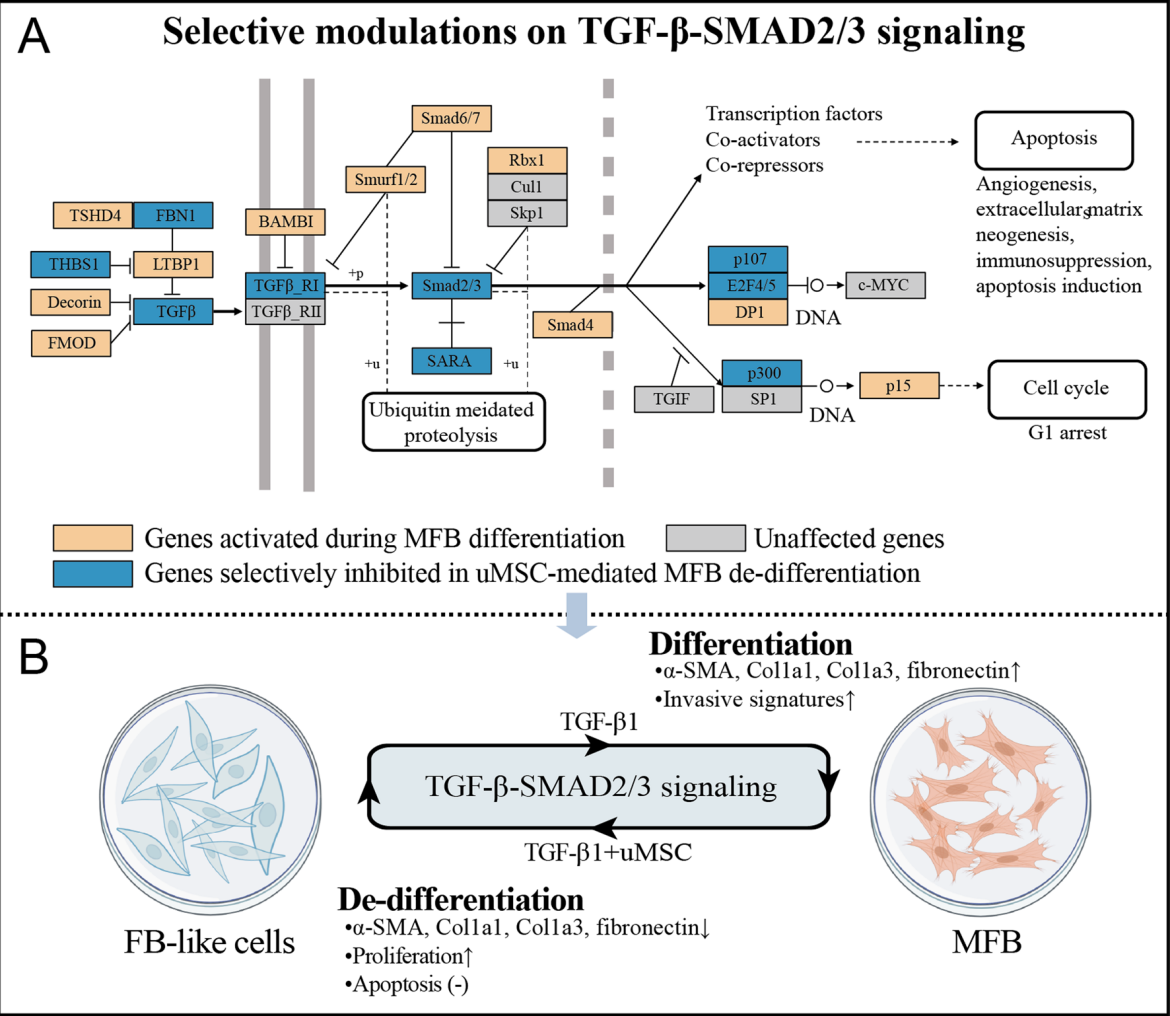
Selective modulations on the TGF-β-SMAD2/3 signaling. **A** Regulations of transcripts in TGF-β-SMAD2/3 signaling according to the KEGG database. **B** uMSC reversibly de-differentiate TGF-β1-induced MFB into a group of FB-like cells by modulating the TGF-β-SMAD2/3 pathway

To further validate this uMSC-mediated regulation, RT-qPCR assays on the core transductors of TGF-β-SMAD2/3 signaling were performed on the FB-derived progenies from each culture group setting. In accordance with the dynamics of those MFB-specific markers during culturing processes (Fig. 5C), results from RT-qPCR assays confirmed a significantly up-regulated expression of TGF-β-SMAD2/3 transductors (*TGFB1*, *TGFBR1*, *SMAD2,* and *SMAD3*) in MFB compared with FB and significantly down-regulation of these transductors in the de-differentiated FB-like cells (Fig. 7E). Short-term and long-term co-culture with uMSC led to durable inhibition of the TGF-β1-induced expression of TGF-β-SMAD2/3 transcripts (Fig. 7E, group 3 and 4). Importantly, plasticity was also noted concerning the activation status of the TGF-β-SMAD2/3 pathway in MFB. The expression of these TGF-β-SMAD2/3 transcripts was once again elevated after the withdrawal from the uMSC feeder layer (Fig. 7E, group 5). At last, by integrating the dynamics of TGF-β-SMAD2/3 transcripts into the Kyoto Encyclopedia of Genes and Genomes Atlas (KEGG), detailed modification of the TGF-β-SMAD2/3 signaling in uMSC-mediated MFB de-differentiation was revealed (Fig. 8A). Together, these results from RNA-seq and in vitro assays revealed that uMSC cells reversibly de-differentiate TGF-β1-induced MFB into a group of FB-like cells by modulating the TGF-β-SMAD2/3 pathway, with a boosted proliferation seen in these cells. These cells largely resemble FBs at the transcriptome level yet exhibit unique surface antigen expression and functional signatures (Additional file 1: Fig. S3). These FB-like cells are still sensitive to TGF-β1 and could immediately re-induced into MFB after the withdrawal of the uMSC feeder layer (Fig. 8B).

## Discussion

The initiation of FMT and the excess accumulation of MFB are considered pivotal pathological events during the development of small airway fibrosis (Bergeron et al. 2013; Neuringer et al. 1998; Kelly et al. 2012; Yang et al. 2020). Functional phenotypes of MFB could not be long-term maintained in the absence of pro-fibrotic cytokines at an ex vivo state (Kuhn and McDonald 1991). Therefore, the lack of models capable of simulating local lung microenvironments largely hinders revealing the FMT under pathological conditions. In the first part of this study, we took IPF as a paradigm to investigate molecular/functional entities during the FMT. The hypertension of TGF-β1 and its downstream pathways are considered landmarks of lung fibrosis (Saito et al. 2018). Along with the activation of muscle tissue formation, our bioinformatic analysis on GEO datasets confirmed a prominent signature of response to TGF-β1 in these patient-derived FB samples. In accordance with previous observations, our data confirmed that administration of TGF-β1 on normal FB readily initiated the pro-fibrotic FMT from both transcriptional and functional levels. These findings provided a rationale for the administration of TGF-β1 to simulate FMT in vitro, which could be employed for further studies on regulations of uMSC to the FMT.

According to published literature, the potential modulatory effect on MFB is more likely to rely on the secretion of a spectrum of MSC-derived soluble factors and subsequent downstream signal transductions within MFB. These identified soluble factors vary to a great extent across studies, including condition medium (Filidou et al. 2022; Zhang et al. 2015), exosomes (Hu et al. 2020) and a spectrum of microRNA (Qiu et al. 2022; Basalova et al. 2020; Fang et al. 2016) (Additional file 1: Table S3). By constructing in vivo and in vitro models simulating fibrotic disease, these MSC-derived factors have been revealed to block/reverse MFB differentiation and/or alleviate fibrotic phenotypes in animal models. Collaborating with these observations, our TGF-β1-induced FMT model and non-contact uMSC co-culture model confirmed that uMSCs reverse the expression of TGF-β1-induced MFB markers with down-regulated MFB-associated functions, thus supporting a potential de-differentiation effect of uMSC. Since the main objectives of existing research are to explore the core transductors and to reveal the reversibility of uMSC-mediated MFB dedifferentiation, we did not explore the secretory properties of uMSCs.

Given the fact that FB, MFB and MSC share a common mesenchymal origin, we next ruled out the possibility of a potential assimilation effect by confirming the lack of MSC-specific transcripts in these FB-like cells (Additional file 1: Fig. S2). However, our further analysis revealed that these FB-like cells still showed certain differences at the transcriptome level compared with untreated FB (Additional file 1: Fig. S3). Therefore, these data revealed that uMSC de-differentiates TGF-β1-induced MFB into a group of FB-like cells.

Recent studies suggest that MSC may also serve as a potential promoter of local fibrosis under certain circumstances (Qin et al. 2023). This duality of MSC regulations on fibrosis could be attributed to the potential "domestication" effect of MSC in response to the pro-inflammatory (high TNF-a, IL-1b) and oxidative (high ROS) local environment, which is well characterized in IPF and BO tissues (Wang and Yang 2022). Exposure to pro-inflammatory cytokines (etc., TNF-a, IL-1b and IL-6) and reactive oxygen species (ROS) potentially induced senescence-associated secretory phenotype (SASP) and senescence in MSCs (Wallace and Friedman 2014; Shenderov et al. 2021; Yao et al. 2020), thus facilitating them into a pro-fibrotic phenotype with the elevated secretion of pro-fibrotic cytokines. Our additional analysis of the secretory functions of MSCs (Rubinstein-Achiasaf et al. 2021) further confirmed that exposure to a pro-inflammatory microenvironment readily induced SASP and up-regulated expression of pro-fibrotic cytokines, including IL-6, IL-7, IL-1A and FGF2 (Additional file 1: Fig. S4). Collaborating with these observations, although our present study confirmed that uMSCs could suppress the MFB phenotype in vitro and de-differentiate them into FB-like cells, existing data also highlight the potential pro-fibrotic properties of allogeneic MSCs. Demonstrated by the significantly boosted proliferation of these de-differentiated FB-like cells, it is reasonable to speculate that the introduction of allogenic MSC would amplify the TGF-β1-sensitive FB-like cells, which might potentially serve as a “workshop” of pro-fibrotic MFB under a TGF-β1 abundant microenvironment. These data also potentially explain the non-durable efficacy of allogeneic MSC therapy in animal models as well as BO patients (Wan et al. 2020; Wu et al. 2015; Ni et al. 2018). However, the responsiveness of allogeneic MSCs to the host microenvironment, and the persistence of the resulting MFB de-differentiation remain largely unexplored.

Despite the extremely varied spectrum of MSC-associated secretory factors (such as numerous kinds of miRNAs) involved in the MFB regulations, the downstream signal transductions during the MFB de-differentiation process remained undetermined. Proposed regulatrory pathways include the TGF-β-SMAD2/3 signaling (Hu et al. 2020; Fang et al. 2016; Wang 2023), WNT/b-catenin signaling (Zhang et al. 2015) and PI3K/Akt, MAPK signaling (Li et al. 2020) and PD-1/PD-L1 signalting (Ni et al. 2018). Revealed by multidimensional transcriptome analysis, our current results indicated that both the initiation of MFB differentiation and uMSC-mediated dedifferentiation was prominently correlated with the activation/inhibition of TGF-β-SMAD2/3 signaling. Most of the TGF-β1-induced transductors (TGF-β1, TGFBR1, and SMAD2/3) in TGF-β-SMAD2/3 signaling by TGF-β1 treatment were rescued after co-culturing with uMSC. However, the canonical/non-canonical WNT signaling was insignificant in these regulations. These observations were consistent with the previous report by Hu et al. (2020) which reveals a significant suppression of FMT by inhibiting the TGF-β-SMAD2/3 signaling.

Although our current data and previous reports have shown that MSC cells have the potency to block and regulate pathological FMT processes, their vulnerability to microenvironmental stress is one major hindrance in treating BO and other fibrotic diseases (Gao et al. 2022). Our demonstration of the reversibility of the uMSC-mediated de-differentiation of MFB and the sensitivity of de-differentiated FB-like cells may partially explain the transient duration of MSC-associated efficacies in the lung fibrotic disease model. However, there are certain flaws in our present study. Since the main objectives of existing research are to explore the core transductors and to reveal the reversibility of uMSC-mediated MFB dedifferentiation, we did not explore the secretory properties of uMSCs. Identifying the functional exosomal properties (e.g., microRNA, lncRNA) could help develop MSC-based cell-free therapies, which may potentially overcome this domestication effect by a pro-fibrotic microenvironment (Hu et al. 2020). On the other hand, targeting the pro-fibrotic microenvironment may be of great therapeutic value in treating established fibrosis.

## Conclusion

To summarize, our findings reported for the first time the reversibility of uMSC-mediated de-differentiation on MFB to FB-like cells by selectively inhibiting the TGF-β-SMAD2/3 signaling. These proliferation-boosted FB-like cells remained sensitive to TGF-β1 and could be re-induced into MFB. The reversible de-differentiation on MFB may explain MSC's inconsistent clinical efficacies in treating BO and other fibrotic diseases. Our work further emphasizes the importance of correcting the pro-fibrotic microenvironment. The potential risk of the augment of TGF-β1-sensitive FB-like cells should be fully considered.

## Supplementary Information

Additional file 1: Figure S1. Flow cytometry assays for the expression of surface antigens CD105, CD73, CD90, CD45, CD34 and HLA-DR. Figure S2. Comparative analysis of the MSC-specific markers and transcripts in normal bone marrow MSC, untreated FB, MFB and FB-like cells.Normalization of potential batch effects using the SVA methods.Normalized mRNA expressionof MSC-specific surface antigens in normal bone marrow MSC, untreated FB, MFB and FB-like cells.Normalized mRNA expressionof osteogenic transcripts in normal bone marrow MSC, untreated FB, MFB and FB-like cells.Normalized mRNA expressionof secretory hematopoiesis-supporting factors in normal bone marrow MSC, untreated FB, MFB and FB-like cells. Figure S3. Differential analysis of transcriptome signatures between FB-like cells and untreated FB.Volcano plot displaying identified DEGs in FB-like cells compared with untreated FB by log2 foldchangeand adjusted P-value. A total of 840 DEGs were identified.Unsupervised clustering heatmap of the transcriptomic level of 21 FB/MFB-associated characteristic transcripts in FB-like cells and untreated FB.Validations of the expression of ACTA2, COL1A1, COL3A1 and FN1 in FB-like cells and untreated FB.KEGG annotations based on significantly up-regulated DEGsand significantly down-regulated DEGsin FB-like cells.Whole transcriptome-based GSEA analysis revealing a signature of inhibited TGF-β1 signaling pathway in FB-like cells. Figure S4. Exposure to pro-inflammatory cytokines induced SASP and up-regulated expression of pro-fibrotic cytokines in normal MSCs.Normal bone marrow MSCs were short-term/long-termcultured with or without pro-inflammatory cytokinesbefore being subjected to RNA-seq. Unsupervised clustering heatmap of the transcriptomic level of 30 SASP-associated cytokines in long-term cultured untreated MSC, long-term cultured treated MSC, short-term cultured untreated MSC, and short-term cultured treated MSC.Normalized mRNA expressionof pro-fibrotic cytokines of MSCs from each culture setting. Table S1. Details of included online datasets. Table S2. The primer and probe sequences for RT-qPCR assays. Table S3. Existing reports on MSC-mediated regulations on MFB.

## Data Availability

The datasets used or analyzed during the current study are available from the corresponding authors upon reasonable request. RNA-seq data in the present manuscript was uploaded to the GEO database (https://www.ncbi.nlm.nih.gov) with the track ID (GSE225159).
