# Supplementary material for: Mesenchymal stem cells reversibly de-differentiate myofibroblasts to fibroblast-like cells by inhibiting the TGF-β-SMAD2/3 pathway
Source: Mol Med. 2023 Apr 25;29:59. doi: 10.1186/s10020-023-00630-9 (PMC10131436; doi:10.1186/s10020-023-00630-9)
Supplement: Supplementary file 1 — Additional file 1: Figure S1. Flow cytometry assays for the expression of surface antigens CD105, CD73, CD90, CD45, CD34 and HLA-DR. Figure S2. Comparative analysis of the MSC-specific markers and transcripts in normal bone marrow MSC, untreated FB, MFB and FB-like cells.Normalization of potential batch effects using the SVA methods.Normalized mRNA expressionof MSC-specific surface antigens in normal bone marrow MSC, untreated FB, MFB and FB-like cells.Normalized mRNA expressionof osteogenic transcripts in normal bone marrow MSC, untreated FB, MFB and FB-like cells.Normalized mRNA expressionof secretory hematopoiesis-supporting factors in normal bone marrow MSC, untreated FB, MFB and FB-like cells. Figure S3. Differential analysis of transcriptome signatures between FB-like cells and untreated FB.Volcano plot displaying identified DEGs in FB-like cells compared with untreated FB by log2 foldchangeand adjusted P-value. A total of 840 DEGs were identified.Unsupervised clustering heatmap of the transcriptomic level of 21 FB/MFB-associated characteristic transcripts in FB-like cells and untreated FB.Validations of the expression of ACTA2, COL1A1, COL3A1 and FN1 in FB-like cells and untreated FB.KEGG annotations based on significantly up-regulated DEGsand significantly down-regulated DEGsin FB-like cells.Whole transcriptome-based GSEA analysis revealing a signature of inhibited TGF-β1 signaling pathway in FB-like cells. Figure S4. Exposure to pro-inflammatory cytokines induced SASP and up-regulated expression of pro-fibrotic cytokines in normal MSCs.Normal bone marrow MSCs were short-term/long-termcultured with or without pro-inflammatory cytokinesbefore being subjected to RNA-seq. Unsupervised clustering heatmap of the transcriptomic level of 30 SASP-associated cytokines in long-term cultured untreated MSC, long-term cultured treated MSC, short-term cultured untreated MSC, and short-term cultured treated MSC.Normalized mRNA expressionof pro-fibrotic cytokines of MSCs fr [file 10020_2023_630_MOESM1_ESM.docx]

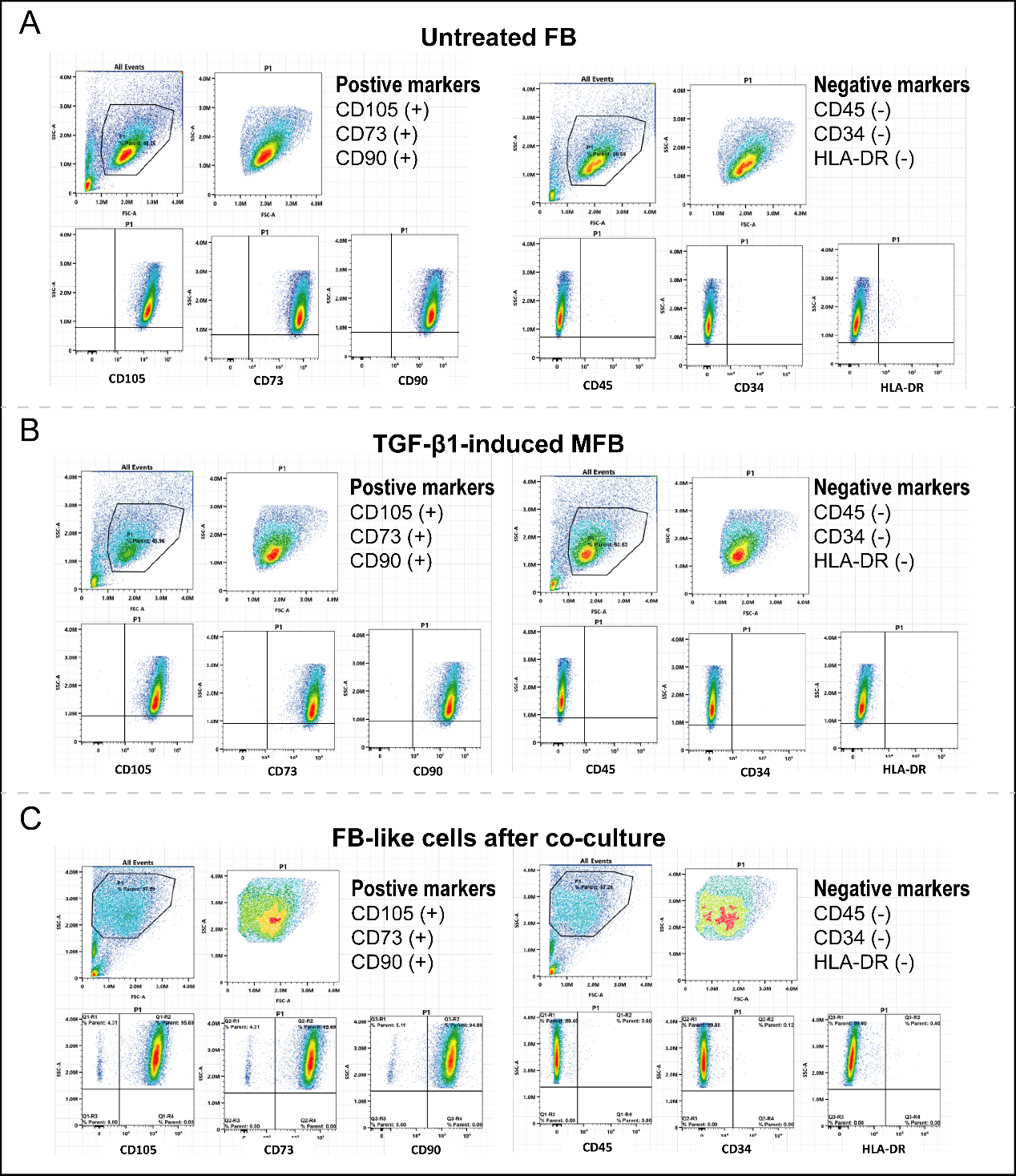


**Figure S1. Flow cytometry assays for the expression of surface antigens CD105, CD73, CD90, CD45, CD34 and HLA-DR**


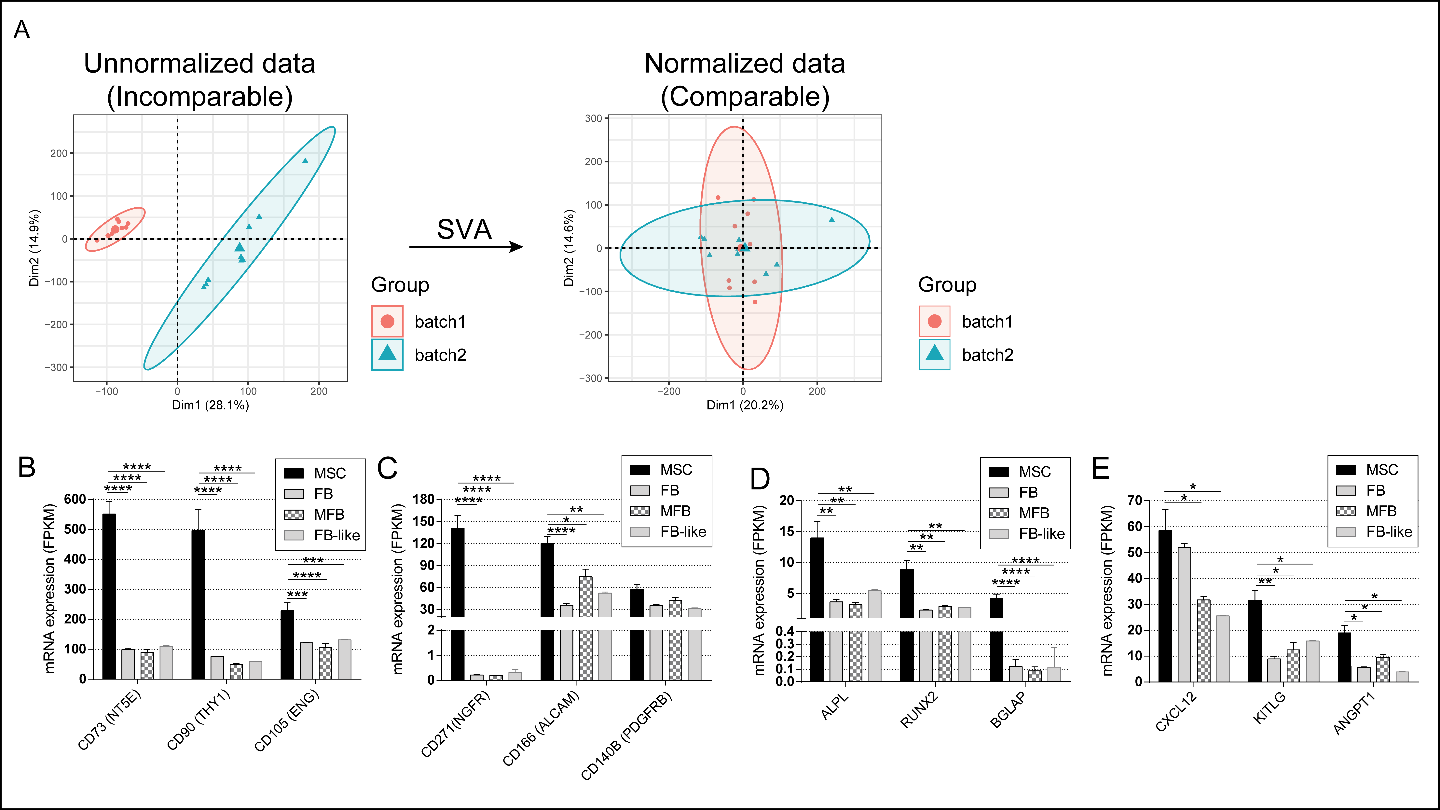
**Figure S2. Comparative analysis of the MSC-specific markers and transcripts in normal bone marrow MSC, untreated FB, MFB and FB-like cells.**

(A) Normalization of potential batch effects using the SVA methods.

(B, C) Normalized mRNA expression (FPKM) of MSC-specific surface antigens in normal bone marrow MSC, untreated FB, MFB and FB-like cells.

(D) Normalized mRNA expression (FPKM) of osteogenic transcripts in normal bone marrow MSC, untreated FB, MFB and FB-like cells.

(E) Normalized mRNA expression (FPKM) of secretory hematopoiesis-supporting factors in normal bone marrow MSC, untreated FB, MFB and FB-like cells.


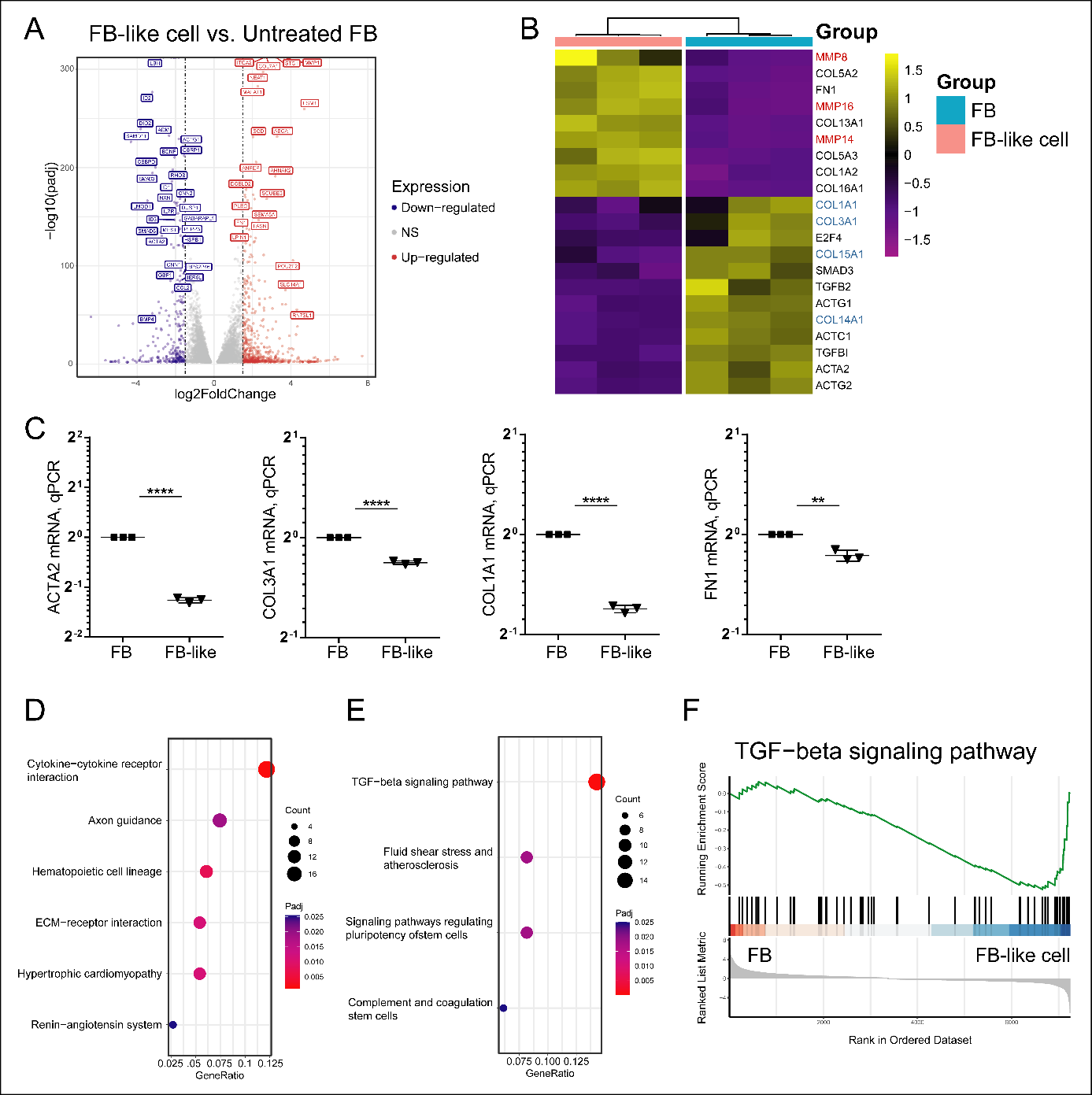
**Figure S3. Differential analysis of transcriptome signatures between FB-like cells and untreated FB**

(A) Volcano plot displaying identified DEGs in FB-like cells compared with untreated FB by log2 foldchange (x-axis) and adjusted P-value (y-axis). A total of 840 DEGs were identified (FB-like cells vs. Untreated FBs; 576 up-regulated and 264 down-regulated).

(B) Unsupervised clustering heatmap of the transcriptomic level of 21 FB/MFB-associated characteristic transcripts in FB-like cells and untreated FB.

(C) Validations of the expression of ACTA2, COL1A1, COL3A1 and FN1 in FB-like cells and untreated FB.

(D, E) KEGG annotations based on significantly up-regulated DEGs (d) and significantly down-regulated DEGs (e) in FB-like cells.

(F) Whole transcriptome-based GSEA analysis revealing a signature of inhibited TGF-β1 signaling pathway in FB-like cells.


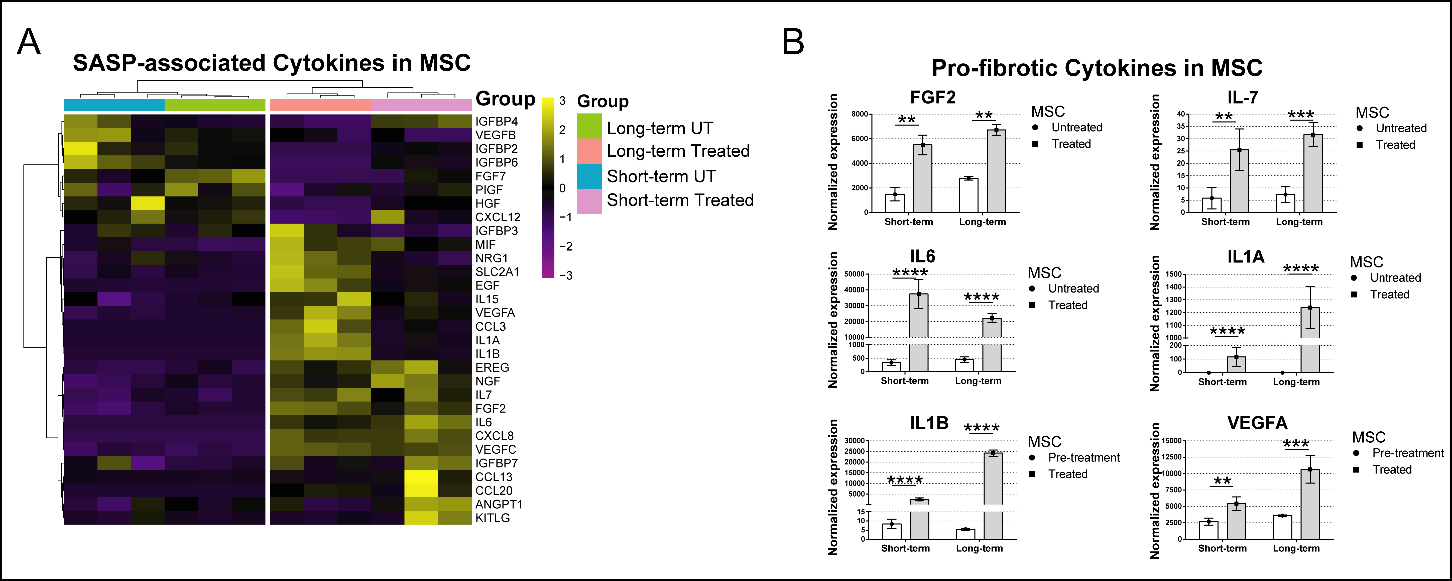
**Figure S4. Exposure to pro-inflammatory cytokines induced SASP and up-regulated expression of pro-fibrotic cytokines in normal MSCs**

(A) Normal bone marrow MSCs were short-term (48h)/long-term (14d) cultured with or without pro-inflammatory cytokines (TNF-a and IL-1b) before being subjected to RNA-seq (GSE161761). Unsupervised clustering heatmap of the transcriptomic level of 30 SASP-associated cytokines in long-term cultured untreated MSC (long-term UT), long-term cultured treated MSC (long-term treated), short-term cultured untreated MSC (short-term UT), and short-term cultured treated MSC (short-term treated).

(B) Normalized mRNA expression (FPKM) of pro-fibrotic cytokines of MSCs from each culture setting.

**Table S1. Details of included online datasets**

| Contributor (s) | Year | Cohorts | FB Samples (n=51) | Sequencing platform | GEO accession |
| --- | --- | --- | --- | --- | --- |
| Deng Z et al. | 2021 | IPF  HD | Apex lung (n=6)  Basal lung (n=6)  Apex lung (n=6)  Basal lung (n=6) | Illumina NovaSeq 6000 | GSE185492 |
| Zhu L et al. | 2021 | IPF  HD | Upper lobe (n=5)  Upper lobe (n=4) | Illumina HiSeq 4000 | GSE180415 |
| Geng Y et al. | 2019 | IPF | Invasive FB (n=9)  Non-invasive FB (n=9) | Illumina HiSeq 3000 | GSE118933 |
| Rubinstein-Achiasaf et al. | 2021 | Human primary bone marrow MSC | Short-term stimulated MSC (n=3)  Short-term untreated MSC (n=3)  Long-term stimulated MSC (n=3)  Long-term untreated MSC (n=3) | Illumina HiSeq 2500 | GSE161761 |

Abbreviations: IPF, idiopathic pulmonary fibrosis; HD, healthy donor; FB, fibroblast; GEO, gene expression omnibus; MSC, mesenchymal stem cell.

**Table S2. The primer and probe sequences for RT-qPCR assays**

| Gene | Forward（5 '-3 '） | Reverse（5 '-3 '） |
| --- | --- | --- |
| *GAPDH* | GGAGCGAGATCCCTCCAAAAT | GGCTGTTGTCATACTTCTCATGG |
| *ACTA2* | TGGCTATTCCTTCGTTACTACTGCT | CATCAGGCAACTCGTAACTCTTCTC |
| *COL1A1* | GAGGGCCAAGACGAAGACATC | CAGATCACGTCATCGCACAAC |
| *COL3A1* | GGAGCTGGCTACTTCTCGC | GGGAACATCCTCCTTCAACAG |
| *FN1* | CGGTGGCTGTCAGTCAAAG | AAACCTCGGCTTCCTCCATAA |
| *TGFB1* | CAATTCCTGGCGATACCTCAG | GCACAACTCCGGTGACATCAA |
| *TGFBR1* | GGCAGAGCTGTGAAGCCTTG | CCTAGCTGCTCCATTGGCAT |
| *SMAD2* | TCATAGCTTGGATTTACAGCCAG | TTCTACCGTGGCATTTCGGTT |
| *SMAD3* | TGGACGCAGGTTCTCCAAA | CCGGCTCGCAGTAGGTAAC |

**Table S3. Existing reports on MSC-mediated regulations on MFB**

| **Authors** | **Source of MSC** | **Disease model** | **Intervention** | **Involved pathway/signaling** | **Modulatory effects on MFB** |
| --- | --- | --- | --- | --- | --- |
| **Wang et al.** **(7)** | Rat BM-MSC | Rat AKI model with renal fibrosis | Local injection of MSCs (renal artery) | Down-regulation of TGF-β-Smad2/3 transcripts | Collagen I/II/III, fibronectin↓  Pericyte-myofibroblast transition↓  Fibrosis score↓ |
| **Qiu et al.****(4)** | Human uMSC | TGF-β1-induced renal myofibroblasts (HK-2 cell line) | In vitro administration of uMSC-exo | miRNA-335-5p binding to ADAM19 | Epi to myofibroblasts transition↓  Inflammation markers↓ |
| **Filidou et al.****(1)** | Immortalized human adipose-derived MSC (aMSC) cell line | Patient-derived pulmonary subepithelial myofibroblast | In vitro administration of aMSC-CM | - | IL-1a↓  IL-1b, IL-6, fibronectin, total collagen↑  Collagen III↓, migration rate↓ |
| **Zhang et al.(2)** | Human uMSC | Irradiation-induced human lung myofibroblast | Non-contact co-culture | Down-regulation of WNT/β-catenin transcripts | FRAT1, a-SMA↓ |
| **Li et al.****(8)** | Mouse uMSC | Streptozotocin-induced diabetic mice with renal fibrosis | Intravenous injection of uMSC and in vitro administration of uMSC-CM | Down-regulation of PI3K/Akt and MAPK signaling | Alleviated renal fibrosis  Proliferation↓  MMP2, MMP9↑ |
| **Hu et al.****(3)** | Human uMSC | TGF-β1-induced dermal myofibroblasts | In vitro administration of uMSC-exo | Down-regulation of TGF-β-Smad2/3 signaling | Collagen I/III, a-SMA, Smad2/3 and pSmad2/3↓ |
| **Basalova et al.****(5)** | Immortalized human aMSC cell line (ACS52telo) | TGF-β1-induced dermal myofibroblasts | In vitro administration of uMSC-derived extracellular vesicles | miRNA-21 and miRNA-29c associated binding | ECM↓  Contractility↓ |
| **Fang et al.****(6)** | Human uMSC | Skin-defect mouse model | Intravenous injection of MSC-derived exomes | miRNA-21, -23a, -125b, 145 associated down-regulation of TGF-β-SMAD2 signaling | Scar formation↓  a-SMA, contractility↓  TGF-β1/SAMD2 transcripts↓ |
| **Present study** | Human uMSC | TGF-β1-induced lung myofibroblasts (MRC-5 cell line) | Non-contact co-culture | Reversible down-regulation of TGF-β-SMAD2/3 signaling | a-SMA, fibronectin, collagen I/III↓  Proliferation↑, apoptosis (-)  Reversible de-differentiation of MFB into FB-like cells |
